# Risk factors for malnutrition among school-aged children: a cross-sectional study in rural Madagascar

**DOI:** 10.1186/s12889-019-7013-9

**Published:** 2019-06-17

**Authors:** Hirotsugu Aiga, Kanae Abe, Vonjy Nirina Andrianome, Emmanuel Randriamampionona, Angèle Razafitompo Razafinombana, Toshiyasu Murai, Masahiro Hara

**Affiliations:** 1 Human Development Department, Japan International Cooperation Agency (JICA), 3rd floor, Nibancho Center Building, 5-25 Niban-cho, Chiyoda-ku, Tokyo, 102-8012 Japan; 2 Department of Global Health, Milken Institute School of Public Health, The George Washington University, 950 New Hampshire Ave, NW, 7th floor, Washington, DC 20052 USA; 3 JICA Participatory and Decentralized School Management Support Project, INFP Mahamasina Porte, 215 Antananarivo, Madagascar; 4 Ministry of National Education, Bâtiment Ex-UERP, Immeuble FFM, 1ère étage, porte 115 Bis, Complexe Ampefiloha, 101 Antananarivo, Madagascar; 5 Association des consultants a la recherche au devellopement economique et social (ACREDES), Ministry of National Education, Ambohidrapeto Lot IAB, 21 G Antananarivo, Madagascar; 6 International Support and Partnership for Health (ISAPH), P. O. Box 35, Mzimba, Malawi

**Keywords:** Madagascar, Malnutrition, School-aged children, School feeding

## Abstract

**Background:**

For over 20 years, Madagascar has been challenged by continued high prevalence of stunting, underweight and wasting among children under 5 years of age. Yet, nutritional status of post-under-five age group has never been assessed in the country, despite its importance in relation not only to physical health but also to cognitive capacity and educational achievements of children. This study aims to estimate prevalence of malnutrition among schoolchildren aged 5–14 years in Madagascar. It further attempts to identify the possible risk factors for their malnutrition. This is the first study that estimates prevalence of malnutrition among school-aged children in Madagascar.

**Methods:**

A cross-sectional household survey was conducted in Antananarivo-Avaradrano district, Analamanga region, Madagascar. The study targeted 393 first and second graders 5–14 years of age enrolled at 10 primary schools, where school-feeding was implemented. Data were collected from anthropometric measurements, their subsequent household structured interviews and observations. Bivariate (Chi-square test or Mann-Whitney’s U test) and multivariable (logistic regression) analyses were performed, to identify the possible risk factors associated with malnutrition.

**Results:**

The overall prevalence rates of stunting, underweight and thinness were 34.9%, 36.9% and 11.2%, respectively. Nineteen children (4.8%) suffered from all the three forms of undernutrition. Older schoolchildren had a significantly greater likelihood of being stunted, underweight and thin. The greater number of members a household had, the higher likelihood of being stunted and thin its schoolchild had. Children having lower Household Dietary Diversity Score were more likely to be underweight. Yet, ‘*Had lunch at school yesterday*’ was associated neither with being stunted nor with being underweight and thin. This implies room for improvement of the current school feeding program.

**Conclusions:**

Prevalence rates of stunting and underweight among 393 children examined were as high as the national averages among children under 5 years of age. Adequate food availability and dietary diversity over a sufficient period (incl. 5–14 years of age) are necessary for increasing likelihood of catch-up in height-for-age and weight-for-age, which are expectable during adolescence. To supplement inadequate household dietary diversity practices, school-feeding program may need to use more animal-protein ingredients.

**Electronic supplementary material:**

The online version of this article (10.1186/s12889-019-7013-9) contains supplementary material, which is available to authorized users.

## Background

Nutrition continues to be a key global development agenda under the Sustainable Development Goals (SDGs) after the completion of the Millennium Development Goals (MDGs) era. Despite a critical need for addressing malnutrition among all populations, the monitoring indicators currently in SDG2 and previously in MDG1 are consistently focused on children under 5 years of age [1, 2]. While admitting children under 5 years of age serve not only as the highest priority sub-population but also as a leading proxy for entire populations in monitoring nations’ nutritional status, it is necessary to capture broader age groups. Scaling Up Nutrition (SUN), a global multisectoral movement on nutrition, addresses malnutrition in a more comprehensive manner, by expanding its target monitoring groups beyond children under 5 years of age (children 13–15 years of age, women 15–49 years of age, and adults ≥18 years of age) [3]. Yet, schoolchildren aged 5–14 years remain excluded from the monitoring frameworks of these major global development agendas as if they were a neglected sub-population. As a result, school-aged children are rarely targeted in nutrition surveys, despite significant impacts of nutritional status on their health, cognition, educational achievements and future economic productivity [4, 5].

Malnutrition among children under 5 years of age has been regularly monitored in all countries since the WHO’s launch of the standard growth chart in 1978 [6]. Yet, it was just 2007 when WHO launched the standard growth chart for children and adolescents aged 5–19 years, by applying prevalence of stunting, underweight, and not wasting but thinness as the malnutrition indicators [7]. The long-term absence of the technical tools for monitoring the three malnutrition dimensions until 2007 might have discouraged researchers from conducting studies on malnutrition among school-aged children. Recently, World Bank started highlighting a need for addressing malnutrition among school-aged children as a foundational investment a country should make, by launching the Human Capital Measurement Project [5]. Thus, a global momentum is being created toward more studies on malnutrition among school-aged children.

For over 20 years, Madagascar has been challenged by consistently higher prevalence of stunting among children under 5 years of age (around 50%) for decades. The latest data (49.2% as of 2016) is the fifth highest in the world [8]. To address the country’s long-lasting high malnutrition prevalence, the Government of Madagascar increasingly focuses on multi-sectoral coordination for greater coverage and impacts of the national nutrition program, by launching the third National Nutrition Action Plan (PNAN III). PNAN III also calls for multisectoral interventions for school-aged children, e.g. community-based deworming, school feeding, household food security, and crop diversification [9]. As a subcomponent policy of PNAN III, National School Feeding and Nutrition Strategy (SNANS) specifies the school-based nutrition interventions. Nevertheless, even basic malnutrition indicators among school-aged children (i.e. prevalence of stunting, underweight, thinness, overweight and obesity) have been neither analyzed nor reported. It is generally difficult for stunted children to catch up during 24–59 months of age [10]. Therefore, the malnutrition status among children under 5 years of age in Madagascar implies the equally high malnutrition prevalence among those ≥5 years of age, due to chronic food insecurity and inadequate feeding practices in the country [11].

In view of unknown but presumably higher malnutrition among school-aged children, the Malagasy Ministry of National Education (MoNE), in collaboration with Japan International Cooperation Agency (JICA), has been committed to strengthening the community-based school feeding program, by developing and piloting its effective and sustainable model. Basic framework of the program defines community-based school feeding as regular provision of meals on school days using locally either produced, procured or donated food items through a school management committee. This study provides the program with the baseline data which not only inform its detailed design but also will be compared with end-line data in the future.

This study is aimed at estimating malnutrition prevalence among children enrolled in rural public primary schools in Madagascar, where school-feeding is implemented, as the pre-intervention baseline data. It further attempts to identify the possible risk factors for malnutrition among them. This study is expected to contribute to informing the design of the community-based school feeding program, by making evidence-based recommendations on it.

## Methods

A cross-sectional household survey was conducted, to estimate malnutrition prevalence (i.e. stunting, underweight, thinness, overweight and obesity) among school-aged children in Antananarivo-Avaradrano district, Analamanga region, Madagascar. Note that stunting and underweight among children 5–19 years of age are defined in the same manner as those among children under 5 years of age (i.e. by employing z-scores for height-for-age and weight-for-age). Yet, thinness among children 5–19 years of age is differently defined from wasting among children under 5 years of age, though both are aimed at measuring acute malnutrition in common. Thinness is defined as those having a z-score < − 2 for BMI-for-age, while wasting is defined as those having a z-score < − 2 for weight-for-height [7]. Moreover, overweight and obesity among children 5–19 years of age are defined as those having z-scores > 1 and > 2 respectively for BMI-for-age, while definitions of overweight and obesity among children under 5 years of age are based on z-scores for weight-for-height.

### Study area

Antananarivo-Avaradrano district is a typical mountainous area located in central inland Madagascar, where temperature is relatively stable all year around (minimum 10 °C - maximum 27 °C). Eighty-one percent of population of the district live in rural area. Average household size is 4.9 members per household and the proportion of male-headed households is 80.1% [12]. Locally produced food crops include rice, cassava, maize, sweet potato, beans and potatoes, accounting for more than 95% of cultivated areas [13].

### Study group

It was reported to the MoNE that local-food-based school feeding was implemented at approximately 50 public primary schools in Antananarivo-Avaradrano district. Through conducting document reviews and its subsequent systematic school visits, it was confirmed that 10 of them were actually implementing local-food-based school feeding (yet only on a very limited scale) as of August 2017, i.e. Ambohimarina, Ambohitrarahaba, Ambohitsoa, Ambovona, Ankadinandriana, Isahafa, Ikianja, Soavinandriamanitra, Tsarahasina, and Viliahazo primary schools. Therefore, all 10 primary schools were targeted in the study. The other 40 primary schools were not implementing school feeding, primarily due to relatively poorer schools’ commitments. Of all children enrolled in the 10 schools, exclusively first and second graders were targeted in this study, for the three reasons. First, targeting first and second graders would help minimize the number of dropouts in the post-intervention survey. This is because they will be more contactable at the time of post-intervention survey to be conducted 4–5 years after strengthening local-food-based school feeding starts. They are likely to stay enrolled in the same schools without going on to secondary schools or getting a job elsewhere. Second, a majority of first and second graders have just entered post-under-five age group, in which the likelihood of recovering from stunting might be expected [14, 15]. Third, as *AnthroPlus*, a WHO’s software, allows z-scores for weight-for-age to be calculated only for children up to 120.8 months of age [16], first and second graders who are overall younger than that age should be targeted.

### Sample size and sampling methods

Prevalence of malnutrition (i.e. stunting, underweight, thinness, overweight and obesity) among school-aged children was previously reported neither from Antananarivo-Avaradrano district nor from any other parts of Madagascar. National prevalence of stunting among children under 5 years of age has been around 50% for decades [8]. Therefore, we assumed that the prevalence among first and second graders could be 50%, under which sample size becomes the greatest (i.e. sufficient sample size also for estimating prevalence rates of underweight, thinness, overweight and obesity). To estimate prevalence representative of children 5–14 years of age enrolled in public primary schools implementing local-food-based school feeding in Antananarivo-Avaradrano district, the sample size was calculated with α (error) = 0.05 and *d* (precision) = 0.05. Thus, 384 school-aged children were determined as the required sample size. Assuming 5% of non-response rate [17], 404 children were employed as the final sample size. To ensure equal representativity of children in each stratum (school, grade and sex), probability-proportional-to-size sampling was applied (Table 1) [7, 8, 18–20]. A total of 404 first and second graders were randomly selected from the respective strata, using student registries of the 10 primary schools.

**Table 1:** Prevalence of malnutrition among schoolchildren 5–14 years of age

Primary school	*N*	Stunting^a^ *n* = 393		Underweight^b^ *n* = 382		Thinness^c^ *N* = 393		Overweight^d^ *N* = 393		Obsesity^e^ *N* = 393	
		*n*	(%)	*n*	(%)	*n*	(%)	*n*	(%)	*n*	(%)
Ambohimarina	25	9	36.0	7	30.4	2	8.0	1	4.0	0	0.0
Ambohitrarahaba	106	33	31.1	36	34.6	10	9.4	1	0.9	0	0.0
Ambohitsoa	38	11	28.9	10	27.8	3	7.9	0	0.0	0	0.0
Ambovona	9	5	55.6	5	55.6	3	33.3	1	11.1	0	0.0
Ankadinandriana	21	10	47.6	9	42.9	1	4.5	0	0.0	0	0.0
Isahafa	45	12	26.7	14	31.8	2	4.4	1	2.2	0	0.0
Ikianja	31	13	41.9	12	41.4	5	16.1	0	0.0	0	0.0
Soavinandriamanitra	35	14	40.0	15	45.5	5	14.3	0	0.0	0	0.0
Tsarahasina	50	18	36.0	20	40.0	5	10.0	0	0.0	0	0.0
Viliahazo	33	12	36.4	17	51.5	8	24.2	0	0.0	0	0.0
Total	393	137	34.9	145	36.9	44	11.2	4	1.0	0	0.0
		95% CI: 30.4–39.7		95% CI: 32.2–41.9		95% CI: 8.5–14.7		95% CI: 0.4–2.6		95% CI: 0.0–1.0	
National prevalence among children under 5 years of age		49.2^f^		36.0^g^		(n.a.)^h^		6.2^i^		(n.a.)^h^	

^a^Z-score for height-for-age < −2 [7]

^b^Z-score for weight-for-age < −2 [7]. As WHO’s growth reference does not cover children 121 months of age and older, 11 children categorized into the age group were excluded from estimation of prevalence of underweight [18]

^c^Z-score for BMI-for-age < − 2 [7]

^d^2 > Z-score for BMI-for-age > 1 [7]

^e^Z-score for BMI-for-age > 2 [7]

^f^Nationally representative prevalence among children under 5 years of age as of 2015 (IFPRI, 2016) [8]

^g^Nationally representative prevalence among children under 5 years of age as of 2008–2012 (UNICEF, 2018) [19]

^h^Thinness and obesity are not internationally employed as malnutrition indicators for children under 5 years of age

^i^Nationally representative prevalence among children under 5 years of age as of 2004 (World Bank, 2018) [20]

### Data collection

Anthropometric measurements of sampled first and second graders and structured interviews with their parents were conducted during the period from 20 November to 11 December 2017, early rainy season in Madagascar. Weight and height of children were measured in classrooms of their primary schools by enumerators. Weight measurements were undertaken to the nearest 0.1 kg using the electronic scale for mother/child 150kg x 100g (UNICEF reference model: S0141021). Height was measured to the nearest 0.1 cm, using the UNICEF standard height scale. To precisely calculate age of children, their dates of birth were collected from student registries. Data of food items used for lunch served at schools on the previous day were collected from school feeding program records at each primary school.

Subsequently to data collection at schools, children’s households were visited to collect data of background variables for malnutrition. A total of 21 background variables were employed to examine their associations with malnutrition among school-aged children (Tables 2 and 3). These background variables were selected primarily from those representing three categories of underlying causes of childhood undernutrition (i.e. household food insecurity, inadequate caring and feeding practices, and unhealthy living environment) [21–23]. The questions on those background variables were included in the structured questionnaire (see Additional file 1). Of them, the data on types of drinking water source, types of toilet, presence of soap/ash for handwashing, food storage, and utensil maintenance were collected through enumerators’ observations. The data on other variables were collected through interviews with parents and other household members responsible for food preparation (incl. Children’s food consumption data based on 24-h dietary recall). Household Dietary Diversity Score (HDDS) was further calculated by summing up the number of food groups consumed by the children during last 24 h [21]. Dietary diversity is a qualitative measure of food consumption that reflects household’s access to a variety of foods, and serves as a proxy for both macro- and micro-nutrient adequacy of the diet of individuals [21]. School feeding can not only complement inadequate household dietary diversity by providing children with food items not being often consumed at their households, but also trigger households’ dietary behavior changes [24].

**Table 2:** Bivariate analyses between schoolchildren’s malnutrition and its categorical background variables

Background variable	Stunting (*N* = 393)					Underweight (*N* = 382)					Thinness (*N* = 393)				
	(+) Stunted		(−) Not stunted		*P*-value ^a^	(+) Under-weight		(−) Not underweight		*P*-value^a^	(+) Thin		(−) Not thin		*P*-value^a^
	*n*	(%)	*N*	(%)		*n*	(%)	*n*	(%)		*n*	(%)	*n*	(%)	
v_1_: Sex															
Male	75	54.7	135	52.7	0.751	83	57.2	121	51.1	0.247	25	56.8	185	53.0	0.749
Female	62	45.3	121	47.3		62	42.8	116	48.9		19	43.2	164	47.0	
Total	137	100	256	100		145	100	237	100		44	100	349	100	
v_2_: Diarrhea during last 14 days															
With diarrhea	4	2.9	8	3.1	1.000	5	3.4	7	3.0	0.771	2	4.5	10	2.9	0.632
Without diarrhea	133	97.1	252	96.9		140	96.6	230	97.0		42	95.5	339	97.1	
Total	137	100	256	100		145	100	237	100		44	100	349	100	
v_3_: Took deworming tables at school															
Took	53	38.7	99	38.7	1.000	63	43.2	85	35.9	0.160	16	36.4	136	39.0	0.870
Did not take	84	61.3	157	61.3		82	56.8	152	64.1		28	63.6	213	61.0	
Total	137	100	256	100		145	100	237	100		44	100	349	100	
Major income sources															
Daily job	46	33.6	93	36.7	Ref.	44	30.3	90	37.9	Ref.	16	36.4	122	35.7	Ref.
v_4_: Agriculture or crop sales	35	25.5	83	32.4	0.137	42	29.0	73	30.8	0.731	10	22.7	110	30.9	0.299
v_5_: Seller, trader or commercial business	23	16.8	22	8.6	0.020*	26	17.9	18	7.6	0.003**	7	15.9	38	10.9	0.317
v_6_: Skilled wage labor	9	6.6	19	7.4	0.839	9	6.2	19	8.0	0.551	0	0.0	28	8.0	0.058
v_7_: Unskilled wage labor	12	8.8	14	5.5	0.286	11	7.6	15	6.3	0.687	5	11.4	21	6.0	0.193
v_8_: Small-scale industry or handicraft	8	5.8	18	6.6	0.831	8	5.5	16	6.8	0.671	5	11.4	20	5.7	0.180
v_9_: Livestock or animal sales	1	0.7	4	1.6	0.662	2	1.4	3	1.3	1.000	0	0.0	5	1.4	1.000
v_10_: Others	3	2.2	3	1.2	0.424	3	2.1	3	1.3	0.678	1	2.3	5	1.4	0.512
Total	137	100	256	100		145	100	237	100		44	100	349	100	
v_11_: Land ownership															
Own land for housing or farming	101	73.7	207	80.9	0.123	111	76.6	188	79.3	0.526	33	75.0	275	78.8	0.562
Don’t own land for housing or farming	36	26.3	49	19.1		34	23.4	49	20.7		11	25.0	75	21.2	
Total	137	100	256	100		145	100	237	100		44	100	349	100	
Education attainment of household member responsible for food preparation															
v_12_: Never gone to school	6	4.4	9	3.5	0.783	6	4.1	9	3.8	1.000	0	0.0	15	4.3	0.392
Dropped out from primary school	72	52.5	112	44.1	Ref.	70	48.3	107	45.2	Ref.	22	50.0	163	46.7	Ref.
v_13_: Primary school	49	35.8	100	39.1	0.586	59	40.7	88	37.1	0.516	16	36.4	133	38.1	0.870
v_14_: Secondary school	8	5.8	29	11.3	0.102	10	6.9	26	11.0	0.210	6	13.6	31	8.9	0.282
v_15_: High school	2	1.5	4	1.6	1.000	0	0.0	6	2.5	0.087	0	0.0	6	1.7	1.000
v_16_: University or other higher education	0	0.0	2	0.4	1.000	0	0.0	1	0.4	1.000	0	0.0	1	0.3	1.000
Total	137	100	256	100		145	100	237	100		44	100	349	100	
v_17_: Type of source of water															
Improved type of source of water ^b^	61	44.5	109	42.6	0.749	57	39.3	107	45.1	0.288	19	43.2	151	43.3	1.000
Not improved type of source of water ^c^	76	55.5	147	57.4		88	60.7	130	54.9		25	56.8	198	56.7	
Total	137	100	256	100		145	100	237	100		44	100	349	100	
v_18_: Type of sanitation facility															
Improved type of sanitation facility ^d^	7	5.1	16	6.3	0.622	7	4.8	16	6.8	0.512	3	6.8	20	5.7	0.733
Not improved type of sanitation facility ^e^	132	94.9	240	93.8		138	95.2	221	93.2		41	93.2	329	94.3	
Total	137	100	256	100		145	100	237	100		44	100	349	100	
v_19_: Hand-washing after toilet of household member responsible for food preparation															
Wash hands after toilet	36	26.3	76	29.7	0.558	40	27.6	71	30.0	0.644	17	38.6	95	27.2	0.155
Don’t wash hands after toilet	101	73.7	180	70.3		105	72.4	166	70.0		27	61.4	254	72.8	
Total	137	100	256	100		145	100	237	100		44	100	349	100	
v_20_: Hand-washing before cooking of household member responsible for food preparation															
Wash hands before cooking	33	24.1	66	25.8	0.807	37	25.5	61	25.7	1.000	14	31.8	85	24.4	0.275
Don’t wash hands before cooking	104	75.9	190	74.2		108	74.5	176	74.3		30	68.2	264	75.6	
Total	137	100	256	100		145	100	237	100		44	100	349	100	
v_21_: Hand-washing before eating															
Wash hands before cooking	33	24.1	70	27.3	0.548	36	24.8	66	27.8	0.553	14	31.8	89	25.5	0.367
Don’t wash hands before cooking	104	75.9	186	72.7		109	75.2	171	72.2		30	68.2	260	74.5	
Total	137	100	256	100		145	100	237	100		44	100	349	100	
Rice storage															
Plastic/metal container with cover	40	29.2	106	41.3	Ref.	40	27.5	104	43.9	Ref.	13	29.5	133	38.1	Ref.
v_22_: Plastic/metal container without cover	1	0.7	8	3.1	0.171	3	2.1	5	2.1	1.000	1	2.3	8	2.3	1.000
v_23_: 50 kg rice bag	32	23.4	45	17.6	0.183	28	19.3	46	19.4	1.000	9	20.5	68	19.5	0.842
v_24_: Handbasket bag	35	25.5	48	18.8	0.121	38	26.2	42	17.7	0.053	13	29.5	70	20.1	0.169
v_25_: Plastic bag	19	13.9	24	9.4	0.179	23	15.9	20	8.4	0.030*	5	11.4	38	10.9	1.000
v_26_: None (buy rice only for the day)	9	6.6	22	8.6	0.599	12	8.3	17	7.2	0.695	3	6.8	28	8.0	1.000
v_27_: Others	1	0.7	3	1.2	1.000	1	0.7	3	1.3	1.000	0	0.0	4	1.1	1.000
Total	137	100	256	100		145	100	237	100		44	100	349	100	
Utensil maintenance															
v_28_: In cabinet after cleaning	23	16.8	45	17.6	0.889	23	15.9	44	18.6	0.580	7	15.9	61	17.5	1.000
In open-space after cleaning	69	50.4	123	48.0	Ref.	74	51.0	111	46.8	Ref.	25	56.8	167	47.9	Ref.
v_29_: Covered with cloth after cleaning	28	20.4	47	18.4	0.686	29	20.0	43	18.1	0.684	10	22.7	65	18.6	0.542
v_30_: In open-space without cleaning	17	12.4	41	16.0	0.373	19	13.1	39	16.5	0.463	2	4.5	56	16.0	0.042*
Total	137	100	256	100		145	100	237	100		44	100	349	100	
Meals taken yesterday															
Breakfast, lunch and dinner/supper	135	98.6	254	99.2	Ref.	144	99.3	234	98.8	Ref.	44	100	345	98.8	Ref.
v_31_: Breakfast and lunch	1	0.7	0	0.0	0.349	1	0.7	0	0.0	0.380	0	0.0	1	0.3	1.000
v_32_: Lunch and dinner/supper	1	0.7	0	0.0	0.349	0	0.0	1	0.4	1.000	0	0.0	1	0.3	1.000
v_33_: Only breakfast	0	0.0	2	0.8	0.545	0	0.0	2	0.8	0.528	0	0.0	2	0.6	1.000
Total	137	100	256	100		145	100	237	100		44	100	349	100	
v_34_: Yesterday’s lunch at school															
Had lunch through school feeding program	25	18.2	47	18.4	1.000	24	16.6	47	19.8	0.498	7	15.9	65	18.6	0.836
Did not have lunch at school	113	81.8	209	81.6		121	83.4	190	80.2		37	84.1	284	81.4	
Total	137	100	256	100		145	100	237	100		44	100	349	100	
Food consumption during last 24 h^f^															
v_35_: Cereal	137	100	256	100	(n.a.)	145	100	237	100	(n.a.)	44	100	349	100	(n.a.)
v_36_: White tubers and roots	57	41.6	97	37.9	0.516	59	40.7	92	38.8	0.747	135	38.7	19	43.2	0.624
v_37_: Vegetables	130	94.9	246	96.1	0.608	136	93.8	229	96.6	0.209	41	93.2	335	96.0	0.421
v_38_: Fruits	13	9.5	22	8.6	0.853	14	9.7	20	8.4	0.713	4	9.1	31	3.9	1.000
v_39_: Meats	38	27.7	82	32.0	0.422	33	22.8	82	34.6	0.016*	106	30.4	14	31.8	0.863
v_40_: Eggs	10	7.3	34	13.3	0.093	32	13.5	11	7.6	0.095	41	11.7	3	6.8	0.450
v_41_: Fish and other seafood	27	19.7	54	21.1	0.795	26	17.9	54	22.8	0.300	6	13.6	75	21.5	0.322
v_42_: Legumes, nuts and seeds	59	43.1	109	42.6	1.000	63	43.4	100	42.2	0.832	20	45.5	148	42.4	0.748
v_43_: Milk and milk products	10	7.3	37	14.5	0.041*	11	7.6	36	15.2	0.036*	5	11.4	42	12.0	1.000
v_44_: Oils and fats	134	97.8	247	96.5	0.554	141	97.2	229	96.6	1.000	43	97.7	338	96.8	1.000
v_45_: Sweets	83	60.6	184	71.9	0.024*	93	64.1	168	70.9	0.176	28	63.6	239	68.5	0.499
v_46_: Spices, condiments and beverage	135	98.5	256	100	0.121	143	98.6	237	100	0.143	44	100	347	99.4	1.000

*P < 0.05

** *P* < 0.01

^a^Chi-square test

^b^Improved types of source of water include: (i) piped household water connection; (ii) public standpipe; (iii) protected well/borehole; (iv) Protected spring; and (v) rainwater collection

^c^Not improved types of source of water include: (i) unprotected well; (ii) unprotected spring; (iii) surface water (river, lake, reservoir, dam, canal, irrigation channel); (iv) vendor-provided water (cart, tanker truck); and (v) bottled water

^d^Improved types of toilet include: (i) flush connected to sewerage system (ii) flush connected to a septic tank; (iii) toilet connected to a pit; (iv) improved ventilated latrine; and (v) latrines with slab

^e^Not improved types of toilet include: (i) flush toilet not connected to sewerage/pit; (ii) latrines without slab/open pit; (iii) bucket; (vi) joint installation with other households (public toilet); and (viii) Outdoor defecation (field, forest, bush and river)

^f^Twelve categories of foods are based on food groups for calculating Household Dietary Diversity Score (HDDS) of Food and Agriculture Organization of the United Nations (FAO) [21]

**Table 3:** Bivariate analyses between schoolchildren’s malnutrition and its interval/ratio background variables

Background variable	Stunting (*N* = 393)					Underweight (*N* = 382)					Thinness (*N* = 393)				
	(+) Stunted		(−) Not stunted		*P*-value^a^	(+) Under-weight		(−) Not underweight		*P*-value^a^	(+) Thin		(−) Not thin		*P*-value^a^
	Mean	s.d.	Mean	s.d.		Mean	s.d.	Mean	s.d.		Mean	s.d.	Mean	s.d.	
v_47_: Age [year]	7.6	1.65	7.0	1.05	< 0.001**	7.4	1.12	6.8	0.99	< 0.001**	7.9	1.95	7.1	1.20	0.004**
v_48_: Total number of household members [person]	5.9	1.82	5.2	1.65	< 0.001**	5.8	1.73	5.2	1.66	< 0.001**	6.3	2.03	5.3	1.67	0.002**
v_48_: Access to source of water by foot [min] ^b^	3.3	3.32	3.7	3.28	0.163	3.4	3.41	3.7	3.28	0.205	3.6	2.68	3.6	3.37	0.454
v_50_: Access to the nearest health facility [min]	34.6	14.3	35.9	19.9	0.485	35.6	16.3	35.1	18.7	0.278	39.1	18.4	35.0	18.1	0.173
v_51_: Household dietary diversity score (HDDS) [pt] ^c^	6.1	1.42	6.3	1.27	0.066	6.0	1.35	6.4	1.29	0.021*	6.2	1.49	6.3	1.31	0.851

*P < 0.05

**P < 0.01

^a^Mann-Whitney U test

^b^The number of minutes spent reaching a water source was measured by allowing an enumerator to walk

^c^Household Dietary Diversity Score (HDDS) is the score ranging from 0 (min) to 12 (max), which is calculated by summing up the number of food groups consumed during previous day (24 h) [21]

Interviews were undertaken by asking the questions in Malagasy, the local language of Madagascar. Two repeated household visits were made, when parents and other household members were absent. The questionnaire was completed by the enumerator during an interview, and later field-checked. Eighteen locally recruited skilled enumerators were trained on anthropometric measurement, interviewing and observation, and supervised by four field team leaders.

### Data analysis

The data obtained through household interviews, observations and anthropometric measurements were entered into a microcomputer. By using *AnthroPlus* (WHO, Geneva) [16] and *R for Mac OS X* version 3.2.2 (R Foundation for Statistical Computing, Vienna) [25], z-scores for height-for-age, weight-for-age and BMI-for-age were calculated based on the WHO standard reference population 5–19 years of age [7, 18]. In Madagascar, children under 5 years of age are sometimes enrolled in first or second graders due to the country’s flexible and less enforced age requirements for primary education enrollment. Those aged under 5 years of age were excluded from the analysis, since they are out of the scope of this study. The statistical analyses other than z-score calculations were conducted, by using *SPSS for Windows*, version 22 (IBM/SPSS Inc., Chicago) [26].

Both bivariate and multivariable analyses were undertaken to identify the background variables (independent variables) associated with whether being malnourished (dependent variable). While the dependent variables are dichotomous, the independent variables are composed of interval/ratio variables and categorical variables. Therefore, two types of bivariate analyses were employed. First, the associations between 16 categorical variables and whether being malnourished were examined, using Chi-square test. For five categorical variables composed of three or more categories (‘*Major income sources*’, ‘*Education attainment of household member responsible for food preparation*’, ‘*Rice storage*’, ‘*Utensil maintenance*’, and ‘*Meals taken yesterday*’), dummy variables were created. As a result, 46 categorical variables were created (incl. 24 dummy variables). The category with the greatest frequency was designated as the reference for the dummy variables. Second, the associations between five interval/ratio variables and whether being malnourished were examined, using a non-parametric method (Mann-Whitney’s U test), as data of those variables were expected not to be normally distributed. Thus, a total of 51 background variables (= 46 categorical + 5 interval/ratio variables) were employed.

The background variables significantly associated with being malnourished (*P* <  0.05 in Chi-square test or Mann-Whitney’s U test) were selected as the possible independent variables for multivariable analyses. Prior to applying them to multivariable analyses, multicollinearity between those possible independent variables was examined by assessing variance inflation factor (VIF). Only those with VIF < 10 were selected as the independent variables for multivariable analyses. As the dependent variables were dichotomous (e.g. stunted or not stunted, underweight or not underweight, and thin or not thin), binominal logistic regression was conducted as the multivariable analyses to control potential confounding variables.

### Ethical consideration

The study protocol was submitted to the National Committee for Biomedical Research Ethics, the Malagasy Ministry of Health, for its ethical clearance. Yet, the Committee officially approved the study protocol by exempting it from an ethical clearance process, due to low level invasiveness of the study design (Exemption letter No. 52).

It was found that all the first and second graders at the target 10 primary schools were under 16 years of age, by reviewing the student registries. Therefore, an advanced informed consent to participate in both anthropometric measurements and structured household interviews was obtained in written form from the parents of each sampled schoolchild through teaching staff of the primary schools. In addition, a verbal informed consent to participate in the study was further obtained from the parents or other household members responsible for food preparation upon a household visit, in case they change their minds.

## Results

Of 404 children sampled, five were dropped due to parental refusal or absence at their households despite successful contacts with children at schools. It was found that six of 399 children were under 5 years of age. Thus, those six children were excluded from the analysis. As a result, data collected from 393 children and their household members were analyzed. WHO growth reference does not cover children older than 120.8 months of age in z-score calculation for weight-for-age. Therefore, 11 children categorized into the age group were excluded from estimation of prevalence of underweight [16].

**Fig. 1:**
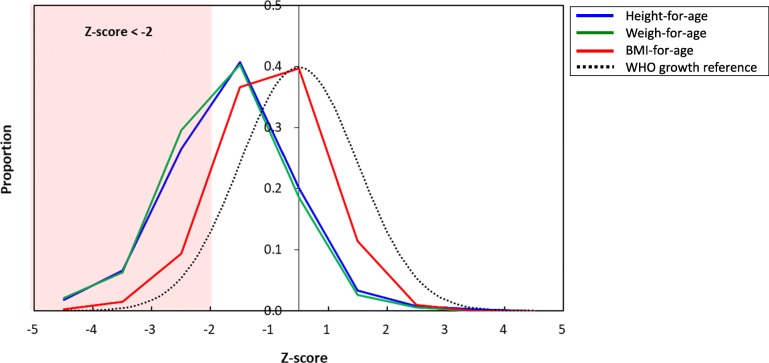
Frequency distributions of z-scores for height-for-age, weight-for-age and BMI- for-age among schoolchildren 5–14 years of age

**Fig. 2:**
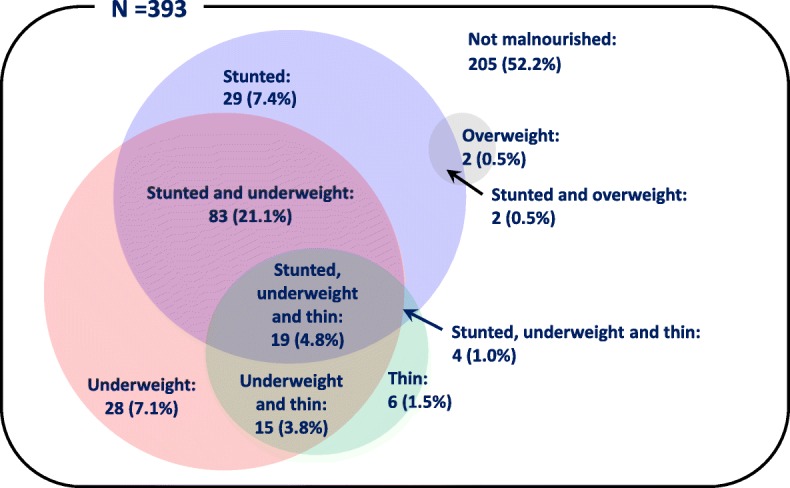
Multiple forms of malnutrition among schoolchildren 5–14 years of age

The overall prevalence rates of stunting, underweight and thinness were 34.9% [95% CI: 30.4–39.7%], 36.9% [95% CI: 32.2–41.9%] and 11.2% [95% CI: 8.5–14.7%], respectively (Table 1 [7, 8, 18–20] and Fig. 1). Of 393 children assessed in this study, 123 (31.2%) suffered from multiple forms of malnutrition (Fig. 2). Note that 19 children (4.8%) suffered from all three forms of undernutrition. Prevalence rates of overweight and obesity were 1.0% [95% CI: 0.4–2.6%] and 0% [95% CI: 0.0–1.0%], respectively. Thus, overnutrition among school-aged children in Antananarivo-Avaradrano district need not be addressed as a major public health issue. For this reason, further analyses were conducted only for stunting, underweight and thinness in search for their possible risk factors.

The background variables were compared between stunted and not stunted children, between underweight and not underweight children, and between thin and not thin children. Of 51 background variables, five produced significant associations (*P* <  0.05) with whether being stunted in bivariate analyses (Tables 2 and 3). Similarly, seven and three background variables produced significant associations (*P* < 0.05) with whether being underweight and with whether being thin, respectively. Moreover, multicollinearity was not confirmed, as only VIF < 10 was detected among them. Therefore, all these background variables with significant associations were employed as the independent variables for logistic regressions.

The results of logistic regressions are shown in Table 4. In the logistic regression on being stunted, four of five independent variables produced significant adjusted odds ratios (ORs) (*P* < 0.05), i.e. ‘*Seller, trader or commercial business*’, ‘*Sweets*’, ‘*Age*’, and ‘*Total number of household members*’. In the logistic regression on being underweight, four of seven independent variables produced significant adjusted ORs (*P* < 0.05), i.e. ‘*Seller, trader or commercial business*’, ‘*Age*’, ‘*Total number of household members*’, and ‘*Household dietary diversity score* ’. In the logistic regression on being thin, two of three independent variables produced significant ORs (*P* < 0.05), i.e. ‘*Age*’ and ‘*Total number of household members*’.

**Table 4:** Logistic regressions on being malnourished with background variables

Logistic regression model	Adjusted OR	95% CI	*P*-value
Logistic regression on being stunted^a^			
v_5_: Seller, trader or commercial business (dummy variable for ‘*Major income sources*’)	2.173	1.120–4.219	0.022*
v_43_: Milk and milk products (dummy variable for ‘*Food consumption during last 24 h*’)	0.671	0.309–1.458	0.313
v_45_: Sweets (dummy variable for ‘*Food consumption during last 24 h*’)	0.616	0.382–0.994	0.047*
v_47_: Age [year]	1.392	1.164–1.664	< 0.001**
v_48_: Total number of household members [person]	1.172	1.031–1.333	0.015*
Logistic regression on being underweight^b^			
v_5_: Seller, trader or commercial business (dummy variable for ‘*Major income sources*’)	2.654	1.339–5.261	0.005**
v_25_: Plastic bag (dummy variable for ‘*Rice storage*’)	1.923	0.962–3.844	0.064
v_39_: Meats (dummy variable for ‘*Food consumption during last 24 h*’)	0.711	0.410–1.233	0.224
v_43_: Milk and milk products (dummy variable for ‘*Food consumption during last 24 h*’)	0.725	0.321–1.634	0.437
v_47_: Age [year]	1.474	1.189–1.827	< 0.001**
v_48_: Total number of household members [person]	1.189	1.043–1.356	0.010*
v_51_: Household dietary diversity score (HDDS) [pt]	0.832	0.701–0.993	0.043*
Logistic regression on being thin^**c**^			
v_30_: In open-space without cleaning (dummy variable for ‘*Utensil maintenance*’)	0.298	0.069–1.284	0.104
v_47_: Age [year]	1.348	1.092–1.663	0.005**
v_48_: Total number of household members [person]	1.240	1.038–1.481	0.018*

*P < 0.05

**P < 0.01

^**a**^The dichotomous independent variable (i.e. stunted or not stunted)

^**b**^The dichotomous independent variable (i.e. underweight or not underweight)

^**c**^The dichotomous independent variable (i.e. thin or not thin)

## Discussion

Prevalence of stunting among children aged 5–14 years (34.9%) was lower than national prevalence of stunting among children under 5years of age (49.2%) [8]. Its data as of 2008–2016, during which the children assessed in this study used to be under 5 years of age, were consistently around 50%, too [18]. Yet, the data specific to Antananarivo-Avaradrano district as of 2008–2016 is not available. We should assume that prevalence of stunting during their under-five childhood was at the same level as the current figure, as catch-up in height-for-age during 24–59 months of age is generally unexpectable [10, 14].

Prevalence of underweight among children aged 5–14 years (36.9%) was at the same level as national prevalence of underweight among children under 5 years of age (36.0%). Prevalence of thinness among children (11.2%) was lower than those of stunting and underweight. Absence of national and provincial prevalence of thinness makes it difficult to compare this result with others. This is because prevalence of thinness has never been reported from Madagascar. Prevalence of thinness is a malnutrition indicator applicable exclusively for 5–19 years of age. Therefore, the indicator is rarely assessed. Note that a majority of nutrition surveys target not school-aged children and older age groups but exclusively children under 5 years of age and their mothers. It is not surprising to learn that the relatively new indicator is generally not attractive enough for health ministries to monitor its data, as it has not been employed as an indicator for global agenda such as SDGs [1].

**Fig. 3:**
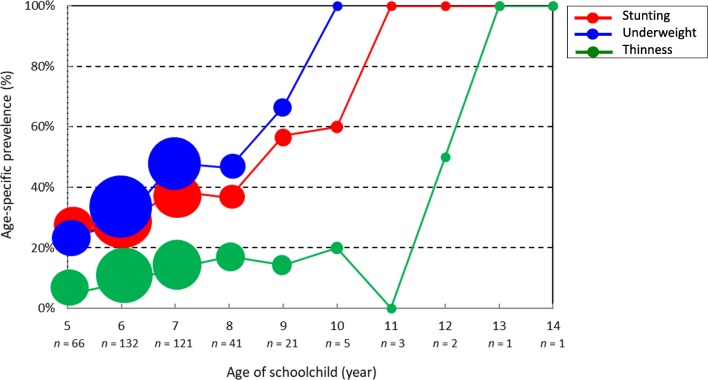
Age-specific prevalence of stunting, underweight and thinness among schoolchildren 5–14 years of age. [Remarks] Sizes of respective plots are in proportion to total number of children

‘*Age*’ produced significant ORs > 1 (*P* < 0.05) in all three logistic regression models for stunting, underweight and thinness (Table 4). This is consistent with the increase in prevalence of stunting, underweight and thinness according to age (Fig. 3). Earlier studies reported that z-score of height-for-age either remains low or even further drops during the period from two to 14 years of age in low- and middle-income countries. It is only adolescence (i.e. 14–20 years of age) when catch-up in height-for-age could occur after the initial 2 years of life [10, 14]. Since there remain 4–5 years for the current first and second graders to enter that age group, impact of strengthening school feeding on height-for-age might not be observable during their primary education enrollments. Nevertheless, adequate food availability and dietary diversity over a sufficient period time including primary education period should be ensured [10, 14]. Thus, strengthening of school feeding will be an essential investment and its evaluation should be conducted not prematurely in a few years but at an appropriate time, i.e. when and/or after first and second graders complete primary education. To increase the likelihood of post-under-two catch-up in both height-for-age and weight-for-age, childhood gastrointestinal infections [14] (schistosomiasis in particular) [27–29] should be adequately prevented and controlled. Madagascar has the world’s fifth highest prevalence of schistosomiasis (52.1% as of 2010) [30]. WHO recommends that all school-aged children be treated by mass praziquantel administration once a year in schistosomiasis high endemic countries with prevalence > 50% [31] such as Madagascar. Though 152 of 399 children (38.1%) assessed in this study underwent school-based deworming, their prevalence of all three forms of undernutrition was not significantly different from those not having undergone it (Table 2). Irregular and sporadic implementations of school-based deworming program might have made deworming less effective. Of 10 primary schools visited, eight implemented deworming interventions before. Yet, at three of eight primary schools, more than 3 years have passed since the last deworming implementation.

Also, ‘*Total number of household members*’ produced significant ORs > 1 (*P* < 0.05) in logistic regression models for stunting, underweight, and thinness (Table 4). This indicates that, the greater number of members a household has, the higher likelihood of being stunted, underweight and thin its schoolchild has. There are two possible reasons for this. First, per-capita food availability at larger households should become smaller. Members of larger households naturally can access and consume a less amount of foods than those of small households, when comparing households at the same income level. In addition, the average number of household members in the lowest quintile income group is the greatest of all quintile income groups, in Madagascar [32]. This synchronously makes it difficult for larger households to ensure per-capita food availability. Second, Malagasy households generally tends to allocate a less amount of foods inadequately meeting age-specific energy requirement to young and older children [33]. Thus, nutritional deficiencies among schoolchildren in this study might be more severe than among their parents. Having assumed this, school-feeding should play a key role as the critical nutrient source supplementary to children’s inadequate diet at household level derived from inappropriate intrahousehold food distribution.

‘*Household dietary diversity score (HDDS)*’ produced significant OR < 1 (*P* < 0.05) in the logistic regression model for underweight (Table 4). This indicates that children consuming a fewer number of food groups are likely to be underweight. Inadequate intake of animal-based protein ingredients (‘*Meats*’, ‘*Milk and milk products*’) were significantly associated with being stunted and underweight (*P* < 0.05) in bivariate analyses (Table 2), though these variables did not produce significant OR (*P* < 0.05) (Table 4). This may imply that there might be a need for improvement of dietary diversity through using particularly animal-based protein ingredients for school meals. Those having ‘*had lunch through school feeding program*’ on the previous day of anthropometric measurements had significantly greater HDDS than those not having (*P* < 0.05: Mann-Whitney U test). Yet, its mean difference between the two groups was only 1.12 (=7.17–6.05). In fact, ‘*had lunch through school feeding program*’ was not associated with being stunted, underweight and thin. These support a need for improvement in school feeding program by increasing the number of food groups in school meals.

This study has limitations in accuracy in schoolchildren’s individual dietary data, as parental 24-h diet recalls and school feeding program records were sources of the data. Yet, monotonous dietary pattern in rural Malagasy households is generally likely to ensure a certain generalizability of 24-h dietary not only over a period of time but also to all household members. Note that parents of 384 children in this study (97.7%) stated that dietary pattern of last 4 days remained the same. Moreover, earlier studies found that parents are reliable reporters at least of their children’s in-home dietary intake [34].

## Conclusions

Prevalence rates of stunting and underweight among 5–14 years of age were as high as those among children under 5 years of age. Difference in the definitions between thinness among children 5–14 years of age and wasting among children under 5 years of age makes it difficult to compare their prevalence rates.

‘*Age*’ and ‘*Total number of household members*’ were identified as the common possible risk factors for stunting, underweight and thinness among schoolchildren aged 5–14 years. Adequate food availability and dietary diversity over a sufficient period time including primary education period should be necessary for increasing likelihood of catch-up expectable during adolescence. Malagasy households have strong preference for rice and cannot afford to buy meats except on the days when they receive income [35]. School feeding program, therefore, should be able to more feasibly procure animal-protein ingredients than households, by leveraging economies of scale in food procurement.

Nevertheless, household feeding practices should improve in parallel, as school feeding can cover only 172 days (Malagasy standard number of school days) out of 365 days (47%) at a maximum in a year. Efforts on feeding practices need to be made both at school and household levels up to 14 years of age and beyond. Note that this study is expected to contribute to evidence-based menu planning for the school feeding program, by serving as the first study that reports risk factors and profiles for malnutrition of school-aged children in Madagascar.

## Additional file

Additional file 1: English version of the questionnaire for the household survey. (PDF 446 kb)

## Data Availability

The datasets used and/or analyzed during the current study are available from the corresponding author on reasonable request.

## Abbreviations

HDDS: Household Dietary Diversity Score
JICA: Japan International Cooperation Agency
MDGs: Millennium Development Goals
MoNE: Ministry of National Education
OR: Odds ratio
PNAN III: The third National Nutrition Action Plan
SDGs: Sustainable Development Goals
SUN: Scaling Up Nutrition
WHO: World Health Organization
