# Supplementary material for: Risk factors for malnutrition among school-aged children: a cross-sectional study in rural Madagascar
Source: BMC Public Health. 2019 Jun 17;19:773. doi: 10.1186/s12889-019-7013-9 (PMC6580631; doi:10.1186/s12889-019-7013-9)
Supplement: Supplementary file 1 — English version of the questionnaire for the household survey. (PDF 446 kb) [file 12889_2019_7013_MOESM1_ESM.pdf]

As of 24 Nov 2017

Form A: HH/Com	Student unique number: #★	
Community…………………………<br>Village: ……………………………………	Date:………(DD)/…………(MM)	Team Code…………………………………<br>Enumerator Code:………………………

Preliminary Feasibility Study for Tafita Madagascar

Questionnaire for:
Children/Households in communities

Informed consent:

Hi, my name is XXXXXX. I would like to request you to spend 30 min responding the questions about your child's schooling and food consumption. If you could kindly collaborate with us, it would be much appreciated. Would you agree to be interviewed and do you allow us to make observations in your house?

☐ (1) Yes; ☐ (2) No ➔ Specify reason……………………………

PART 1: Students information

Q1a★	Name of the school		
Q1b★	Name of the Fokontany		
Q1c★	Name of the child	Family Name	Given Name
Q1d★	Date of birth	…………………(DD) /……………… (MM) /…………………… (YY)	
Q1e★	Sex	1 Male<br>2 Female	
Q1f ▲	Number of school days since 6 November as of date of school visit	☐ days	
Q1g ▲	Number of absent days from school since 6 November 2017	☐ days	
Q1h ▲	Score of recent **Math** test	**Mathematics:**	

1	Beginner
2	Simple addition
3	Simple subtraction
4	2-digit addition and subtraction
5	multiplication
6	3-digit addition and subtraction
7	division

As of 24 Nov 2017

Q1i ▲	Score of recent **Lecture** test	**Lecture:**<br>1 Beginner<br>2 Letter<br>3 Word<br>4 Paragraph<br>5 History
Q1j ●	Distance from home to the school	1 < 30 minute walk<br>2 30 -60 minute walk<br>3 >60 minute walk
Q1k ▲	Height<br>→ Precise at the level of 0.1cm	☐☐☐ . ☐ (cm)
Q1l ▲	Weight<br>→ Precise at the level of 0.1kg	☐☐ . ☐ (kg)
Q1m ●	On any day of the last 14 days, did your child have loose **OR** liquid stool more than 3 times in 24 hr?	1 Yes<br>0 No
Q1n ▲	When did your school conduct mass drug administration of iron tablets?	……………..(DD) /…………….. (MM) /………………. (YY)
Q1o ▲	When did your school conduct mass drug administration of deworming tablets?	……………..(DD) /…………….. (MM) /………………. (YY)

PART2: Household socio-economic information

Q2a ●	Name of the interviewee	**Family name…………………………… Given name……………………………**
Q2b ●	Phone number	Number: …………………………………………………………… 0 No phone
Q2c ●	Relationship between child and Interviewee	1 Father<br>2 Mother<br>3 Grandfather<br>4 Grandmother<br>5 Sister/Brother<br>6 Other (Specify ……………………………)
Q2d ●	How many people are in the household?<br>→ Household is defined as a "Group of persons who live together **and** share income **and** food at least for 6 months"<br>→ Count all the family members one by one	………………………………… (person)
Q2e ●	Major income sources<br>→ Fill the numbers in the right boxes.<br>1: Agriculture and sales of crops<br>2: Livestock and sales of animal<br>3: Fishing<br>4: Unskilled wage labor<br>5: Skilled labor<br>6: Handicrafts/artisanal work<br>7: Seller, commercial activities<br>8: Remittances from families<br>9: Salaries, wages (employee)<br>10: Begging, Assistance<br>11: Government allowance (pension, disability benefit)<br>12: Daily job<br>13: Other (Specify ……………………………)	The greatest income source ☐<br>2nd greatest income source ☐<br>3rd greatest income source ☐

Q2f●	Who are in charge of the greatest source of income	1	Father	4	Grandmother
		2	Mother	5	Other (Specify ……………………………)
		3	Grandfather		
Q2g●	Do the family have their own land?<br>→Land for housing **or** farming **or** renting	1	YES		
		0	No		
Q2h●	Education level of a household member responsible for meal preparation<br><br>→Typically mother of child	1	Never go to any school		
		2	Drop out in Primary school		
		3	Completed Primary school		
		4	Completed Secondary school		
		5	Completed High school		
		6	Completed University and higher education		

PART 3: Household Hygiene Information

Q3a◎	Type of water source for drinking and cooking<br>→See examples in guidelines	**Improved water source**		**Not improved water source**	
		1	Piped household water connection 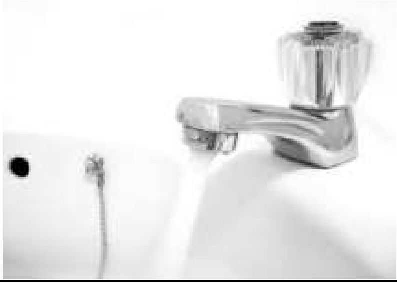	6	Unprotected well 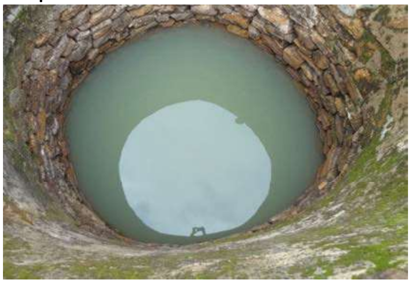
		2	Public standpipe 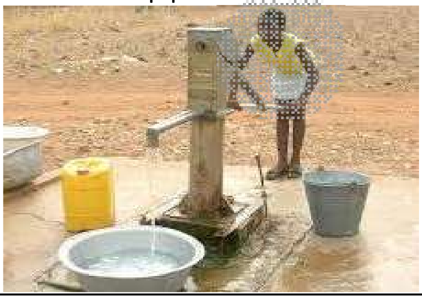	7	Unprotected spring 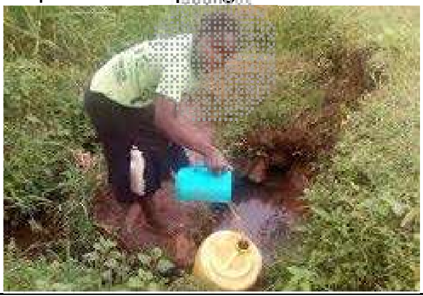
		3	Protected well/borehole 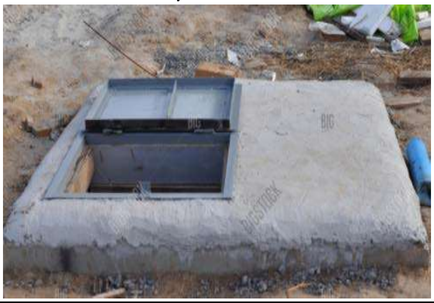	8	Surface water (river, lake, reservoir) 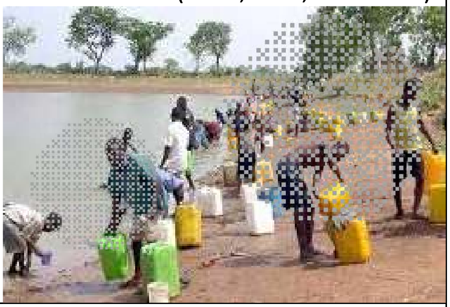
		4	Protected spring 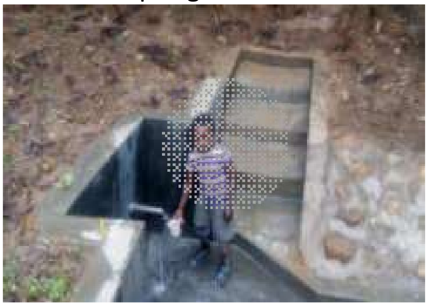	9	Vendor-provided water (cart, truck) 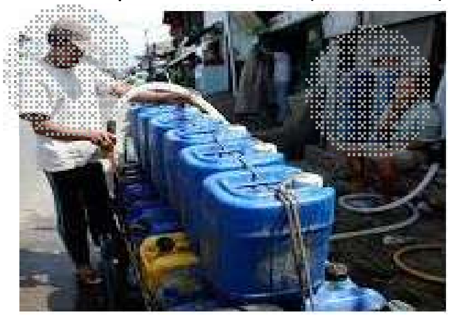

		5	Rainwater collection<br>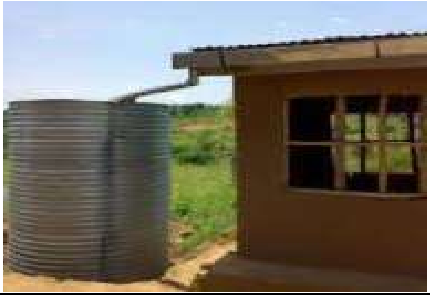	10	Bottled water<br>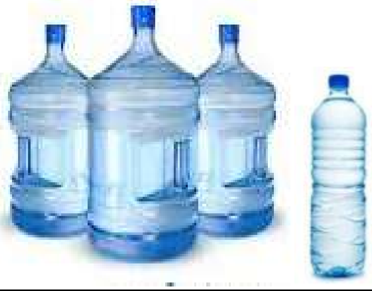
Q3b◎	How long does it take to reach the water source?<br>→Check the watch to estimate how long it takes.				…………………………… (minute)
Q3c◎	Type of toilet<br><br>→See examples in guidelines		**Improved toilet**		**Not improved toilet**
		1	Flush connected to sewerage system<br>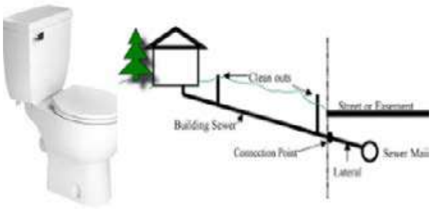	6	Flush toilet not connected to seweage/pit<br>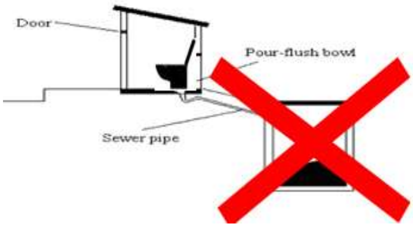
		2	Flush connected to a septic tank<br>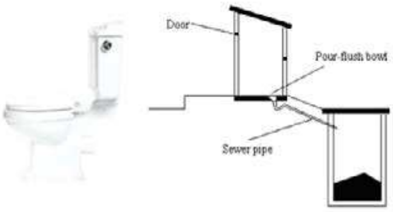	7	Latrines without slab / open pit<br>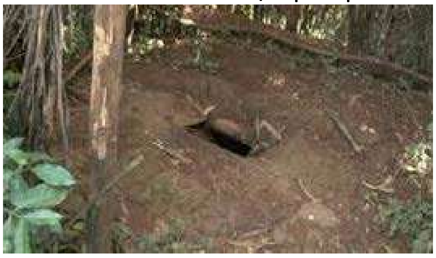
		3	Toilet connected to a pit<br>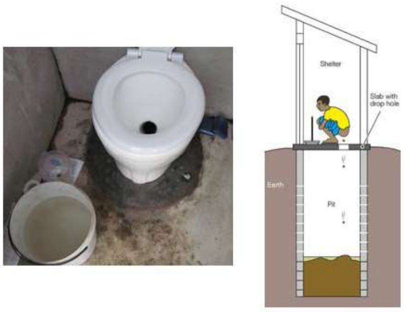	8	Bucket<br>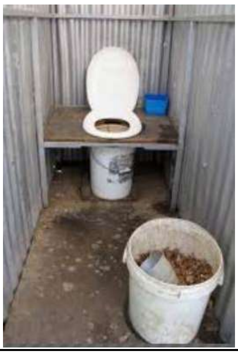
		4	Improved ventilated latrine / Ventilated improved pit (VIP)<br>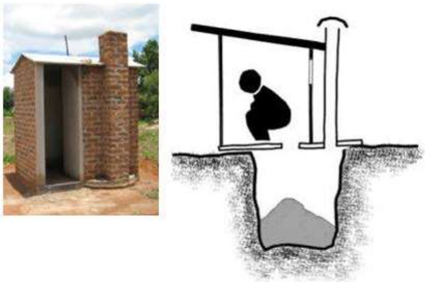	9	Joint installation with other households (public toilet)<br>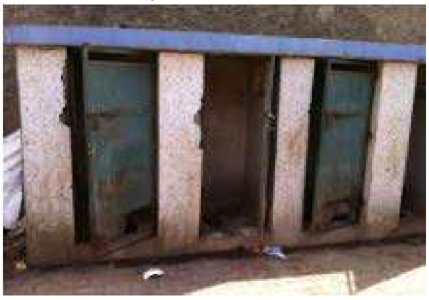

		5	Latrines with slab 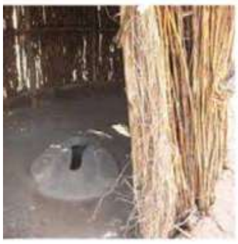 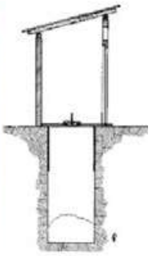	10	Outdoor defecation (field, forest, bush and river) 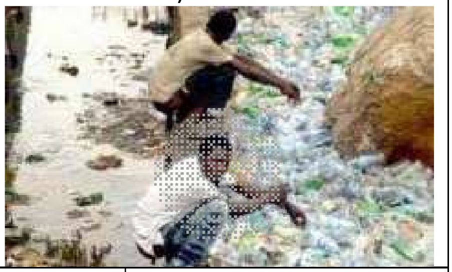

Q3d●	Number of minutes to be spent reaching the nearest health facility?	……………………… minutes	
Q3e●	Wash hands with soap/ash after toilet (to be asked household member responsible for cooking) →Even though soap/ash is not found, no need to re-verify this answer	1<br>0	Yes<br>No
Q3f●	Wash hands with soap/ash before cooking (to be asked household member responsible for cooking) →Even though soap/ash is not found, no need to re-verify this answer	1<br>0	Yes<br>No
Q3g●	Wash hands with soap/ash before eating (to be asked household member responsible for cooking) →Even though soap/ash is not found, no need to re-verify this answer	1<br>0	Yes<br>No
Q3h◎	Presence and status of the soap<br>→ Direct observation	1	Yes, soap wet or seems to have been wet
		2	Yes, soap dried or cracked
		3	Ash
		4	Neither soap nor ash
Q3i◎	Rice storage<br>→ Direct observation	1	Plastic or metal container with cover
		2	Plastic or metal container without cover
		3	50 Kg Rice bag
		4	Other<br>(Specify ……………………………………………….)
Q3j◎	Utensils for eating maintenance (e.g. Spoons, Plates, Soup Bowls)<br>→ Direct observation	1	Storing them in cabinet after cleaning
		2	Storing them in open-space after cleaning
		3	Storing them not in cabinet but covered
		4	Leaving them open-space without cleaning

PART 4: Food Consumption Information

Q4a ▲	Was there school feeding at school yesterday?	1	Yes, breakfast	2	Yes, lunch	3	No

Q4b ▲ If yes, what was the menu?

	**School meals**
**[Example]**	Rice and accompaniment (beans, green leaves…)
**[To be filled]**	

Q4c	Meals taken yesterday from waking up to going to bed (circle all applied)	1	Breakfast
		2	Lunch
		3	Dinner/Supper

Q4d

Diet diversity for 24hr of the previous day (i.e. yesterday)

→ To be completed during the interview

→If target T1/T2 student is present, ask what food he/she ate for snack, which often parents don't know.

	**Breakfast**	**Snack**	**Lunch**	**Snack**	**Dinner**	**Snack**
**[Example]**	Tea, Millet porridge [ground millet; water; sugar]	Ground nuts	Rice, Sauce with greens [cassava leaves; onion; oil]	Mango	Rice, Pumpkin sauce [pumpkin; oil; dried fish; tomato]	Coffee [sugar, milk]

→This information will be converted into Q4e – Q4t. Therefore, it is not necessary to enter the above data into computer.

As of 24 Nov 2017

Food diversity					
Q4e★	[#1] **Cereal:** Corn/maize, rice, wheat, sorghum, millet or any other grains or foods made from these (e.g. bread, noodles, porridge or grain products) + insert local foods e.g. ugali, nshima, porridge or pastes or other locally available grains.	1	YES	0	NO
Q4f★	[#2] **Vitamin A rich vegetables & tubers**: Pumpkin, carrots, squash, sweet potatoes that are orange inside + other locally available vitamin A rich vegetables (e.g. red sweet pepper)	1	YES	0	NO
Q4g★	[#3] **White roots & turbers**: White potatoes, white yams, white cassava, or other foods made from roots	1	YES	0	NO
Q4h★	[#4] **Dark green leafy vegetables**: Dark green/leafy vegetables, including wild ones + locally available vitamin A rich leaves such as amaranth, cassava leaves, kale, spinach, etc.	1	YES	0	NO
Q4i★	[#5] **Other vegetables**: Other vegetables (e.g. tomato, onion, eggplant) including wild vegetables	1	YES	0	NO
Q4j★	[#6] **Vitamin A rich fruits**: Ripe mangoes, cantaloupe, apricots (fresh or dried), ripe papaya, dried peaches + other locally available vitamin A rich fruits	1	YES	0	NO
Q4k★	[#7] **Other fruits**: Other fruits, including wild fruits	1	YES	0	NO
Q4l★	[#8] **Organ meats**: Liver, kidney, heart, or other organ meats or blood -based foods	1	YES	0	NO
Q4m★	[#9] **Flesh meats**: Beef, pork, lamb, goat, rabbit, wild game, chicken, duck, or other birds	1	YES	0	NO
Q4n★	[#10] **Eggs**: Chicken, duck, guinea fowl, or any other egg	1	YES	0	NO
Q4o★	[#11] **Fish**: Fresh or dried fish or shellfish	1	YES	0	NO
Q4p★	[#12] **Legumes, nuts & seeds**: Beans, peas, lentils, nuts, seeds or foods made from them	1	YES	0	NO
Q4q★	[#13] **Milk & milk products**: Milk, cheese, yogurt, or other milk products	1	YES	0	NO
Q4r★	[#14] **Oils & fats**: Oil, fats or butter added to food or used for cooking	1	YES	0	NO
Q4s★	[#15] **Sweets**: Sugar, honey, sweetened soda, sweetened juice or sugary foods such as chocolates, candies, cookies and cakes	1	YES	0	NO
Q4t★	[#16] **Spices condiments & beverages**: Spices (black pepper, salt), condiments (soy sauce, hot sauce), coffee, tea, alcoholic beverages, or local examples	1	YES	0	NO

Q4u★	Regarding the diet diversity of the previous 4 days;<br>Is what you ate yesterday similar to what you have you eaten for 3days before yesterday (4 days ago, 3days ago and the day before yesterday)?	1	**YES**,<br>What I ate yesterday is something similar to what I have eaten 4 days ago, 3 days ago and the day before yesterday.
		0	**NO**,<br>What I ate yesterday is very different from what I have eaten 4 days ago, 3 days ago and the day before yesterday.

LEGEND

★ Data to be transcribed from students' registry at the time of sampling
▲ Data to be calculated and entered by enumerators after school/household visit
▲ Data to be either transcribed or measured at the time of anthropometric measurement
● Data to be collected from interview at households
◎ Data to be collected from observation at households

END
